# Value of transrectal ultrasonography for tumor node metastasis restaging in patients with locally advanced rectal cancer after neoadjuvant chemoradiotherapy

**DOI:** 10.1093/gastro/got028

**Published:** 2013-10-23

**Authors:** Hai-Hua Peng, Kai-Yun You, Cheng-Tao Wang, Rong Huang, Hong-Bo Shan, Jian-Hua Zhou, Xiao-Qing Pei, Yuan-Hong Gao, Bi-Xiu Wen, Meng-Zhong Liu

**Affiliations:** ^1^Department of Radiation Oncology, First Affiliated Hospital of Sun Yat-sen University, Guangzhou, China, ^2^Department of Radiotherapy, Sun Yat-sen University Cancer Center, State Key Laboratory of Oncology in Southern China, Guangzhou, China, ^3^Department of Endoscopy and Laser, Sun Yat-sen University Cancer Center, State Key Laboratory of Oncology in Southern China, Guangzhou, China and ^4^Department of Ultrasonography, Sun Yat-sen University Cancer Center, State Key Laboratory of Oncology in Southern China, Guangzhou, China

**Keywords:** Rectal cancer, neo-chemoradiotherapy (neo-CRT), transrectal ultrasonography (TRUS), TNM restaging

## Abstract

**Objective:** To explore the value of transrectal ultrasonography (TRUS) for tumor node metastasis (TNM) restaging for patients with locally advanced rectal cancer after neoadjuvant chemoradiotherapy (neo-CRT).

**Methods:** One hundred and forty-nine patients with locally advanced rectal cancer (cT3-4 or cN+) who underwent TRUS after neo-CRT were retrospectively reviewed. TRUS restaging was compared with the results of post-operative pathological TNM findings.

**Results:** After neo-CRT, the accuracy of TRUS for diagnosing T-staging was 30.9%, with 60.4% (90/149) of cases overestimated. The sensitivity of TRUS for T-staging (T0 vs T1 vs T2 vs T3 vs T4) were 16.3%, 0%, 12.5%, 42.6% and 75.0%, respectively. The accuracy of TRUS for diagnosing N-staging after neo-CRT was 81.2%, with the sensitivities of N0 and N+ were 93.3% and 31.0%, respectively. After neo-CRT, 27.5% (41/149) of patients achieved pathologically complete response (pCR). The sensitivity, specificity, positive predictive value and negative predictive values of TRUS for pCR were 17.1%, 99.1%, 87.5% and 75.9%, respectively.

**Conclusions:** TRUS can be applied for restaging T4 and N0, and has potential for screening out patients with pCR in those with locally advanced rectal cancer after neo-CRT, although some stages are overestimated for T-staging and its sensitivity for predicting pCR is low.

## INTRODUCTION

Neo-chemoradiotherapy (neo-CRT) is applied in patients with locally advanced rectal cancer, which can make the tumor shrink, reduce the adhesion between tumor and the surrounding organs, improve the resection rate and the rate of sphincter preservation, so as to significantly improve patients' prognosis and quality of life [1, 2]. As a result, neo-CRT has been widely used in the treatment of patients with locally advanced rectal cancer.

In some patients with rectal cancer, the tumor can be significantly reduced after neo-CRT; even the tumor cells entirely disappear by pathologically complete response (pCR). The research shows that the overall relapse-free survival rate and cancer-specific survival rate for patients with pCR are both very satisfactory [3]. It is reported that the curative effect of local resection for patients with locally advanced rectal cancer, obtained pCR after neo-CRT, is also encouraging, which is no less than the effect of radical excision [4, 5]. Methods of carrying out accurate restaging after neo-CRT therapy in patients with rectal cancer—including the assessment of tumor size, depth of tumor invasion and its relationship with surrounding organs, whether it can be completely resected by surgery, or to implement the anal surgery, etc.—is currently a hot research topic.

The accuracy and sensitivity of transrectal ultrasonography (TRUS) are high in evaluating the local infiltration of rectal cancers, making it the first choice of imaging modality for the pre-operative T-staging of rectal cancer [6]. However, it is controversial whether its value and accuracy of TRUS restaging is comparable to pathological restaging when it is used for pre-operative restaging in patients with locally advanced rectal cancer after neo-CRT [7–13]. We explored the accuracy of TRUS for restaging in patients with rectal cancer after neo-CRT and analysed the influence factors in this study by retrospectively comparing the consistency between TRUS for pre-operative restaging after neo-CRT and post-operative pathological staging.

## MATERIALS AND METHODS

This study was approved by SYSU (Sun Yat-sen University) Ethics and Research Committee. The inclusion criteria of this study were as follows: (i) pre-operative endoscopy and pathology biopsy confirmed for the diagnosis of rectum adenocarcinoma; (ii) multiple pre-operative imaging examinations diagnosed as the locally advanced rectal cancer (cT3-4 and/or cN+); (iii) patients accepted the neo-CRT, and performed TRUS exam after neo-CRT and (iv) complete clinical pathological data available. The exclusion criteria were as follows: (i) those who with distant metastasis before neo-CRT confirmed by multiple imaging examinations; (ii) patients combined with other malignant tumors; (iii) history of pelvic radiotherapy or chemotherapy and (iv) patients accepted the emergency surgery.

According to the above standard, 149 consecutive patients with locally advanced rectal adenocarcinoma treated at the cancer prevention and control center of Sun Yat-Sen University from August 2005 to December 2010 were included in this study, including 103 male and 46 female, with an average age of 56 years (range: 15–77 years), 34.9% (52/149) patients with TNM stage II and 65.1% (97/149) of stage III. The median distance of the inferior margin of tumor to the anal verge was 5 cm (range: 1–16 cm). The median value of carcinoembryonic antigen (CEA) before treatment was 3.9 ug/L (range: 0.2–219.8 ug/L). Patients in this study underwent imaging examinations for imaging clinical staging before neo-CRT, including 135 TRUS, 114 computer tomography (CT) and 18 magnetic resonance imaging (MRI). The median time-point for performing TRUS exams after neo-CRT was 35 days (17–61 days), and the median time-point for surgical resection after neo-CRT was 43 days (20–73 days).

### Therapeutic schedule

Three-dimensional conformal radiotherapy technology (3D-CRT) was used for pre-operative radiotherapy, with 6–8 MV X-rays and 3–4 views of isocenter irradiation: the median radiation dose was 46 Gy / 23 fractions. One hundred and forty-four patients received the 46 Gy / 23 times exposure, and five patients accepted the 30 Gy / 10 times exposure.

The simultaneous chemotherapy regimens were FOLFOX or XELOX. Twenty-seven patients received the FOLFOX plan: fluorouracil 3 g/m^2^, CIV lasting for 48 h; calcium folinate 200 mg/m^2^, day 1; oxaliplatin 100 mg/m^2^, day 1; repeated for two weeks. One hundred and nineteen patients received the XELOX plan: capecitabine 1000 mg/m^2^, b.i.d. days 1–14; oxaliplatin 100 mg/m^2^, day 1; repeated for three weeks.

The Miles, Dixon, or Hartmann surgery was performed for all patients with a principle of total mesorectum excision after neo-CRT. The post-operative pathological tissues were assessed in detail, both by gross and microscopic analysis, according to the 2009 version of the AJCC/UICC evaluation criteria, to observe the tumor survival situation and response to the neo-CRT.

The post-operative adjuvant chemotherapy regimens were consistent with the pre-operative chemotherapy scheme, and the median number of adjuvant chemotherapy cycle was 4 (range: 2–6) cycles.

### TRUS exams

The TRUS exams were usually carried out by two experienced attending (or more senior) specialized sonographers. The examination was carried out on Donezhi SSA-790A equipment, which is made in Japan with a special single-probe rectal cavity sonograph normally using frequencies from 8–15 MHz and a 15 cm probe length. In general, preparation for ERUS consists of laxatives and enemas prior to the examination. All patients were placed in a relaxed position on their left sides.

The ultrasonic probe was inserted and advanced toward the upper end of tumor as far as possible. Meticulous observation of (1) the tumor's involvement of rectal wall structure and its relationship to the surrounding tissue and (2) lymphadenopathy was performed by repeatedly changing the probe's orientation.

### TRUS restaging after neo-CRT

According to ultrasonic T-staging (uT) and N-staging (uN) standards, the rectal cancer can be restaged in reference to TRUS after neo-CRT as follows:

- uT-staging [14]
- uT1, the neoplasm invading the rectal mucosa or submucosa
- uT2, the neoplasm invading the rectal muscularis propria
- uT3, the tumor penetrating the muscularis propria to the serosa layer, but not yet through it
- uT4, the mass penetrating the serous membrane layer, and infiltrating the mesentery or adjacent organs (prostate, bladder, vagina, etc.)
- uN-staging [15, 16]
- uN+, the lymph node appears as circular or elliptic with clear margin; low echo or heterogeneous echoes (which is similar to the echoes of primary tumors); and short diameter >5 mm
- uN0, no lymph nodes, or lymph nodes that did not meet the above descriptions.

If the difficulty was encountered in diagnosing the involvement of primary tumor and/or lymphadenopathy after neo-CRT, two or more experts were asked for second opinions. If three experts disagreed with each other, a majority decision was adopted.

### Statistical analysis

All statistical analyses were performed using SPSS 17.0 software. Chi-squared and Fisher's exact test were adopted for the comparison of qualitative data, and *P* values <0.05 were accepted as statistically significant. Indicators such as accuracy, sensitivity and specificity were used to evaluate the diagnostic values of TRUS.

## RESULTS

### The comparison of TRUS staging with post-operative pathological staging

**Fig. 1.**
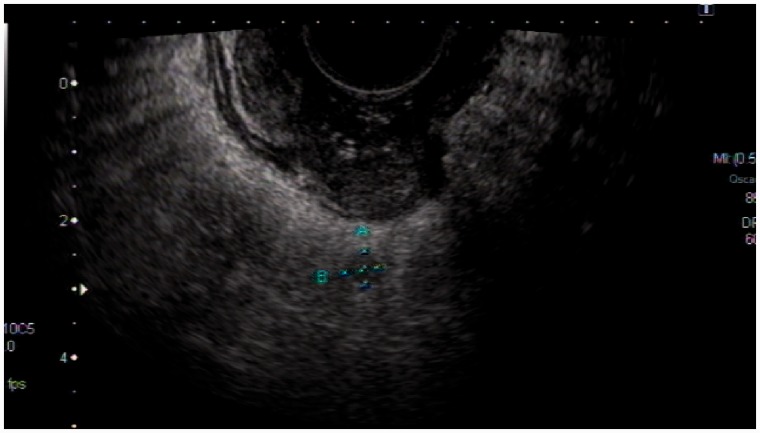
The neoplasm infiltrated through the intestinal wall and invaded to the serosa layer, with a hypoecho node of 0.5 cm diameter; TRUS restaged for uT4N1. The post-operative pathology staged for ypT4N1.

**Fig. 2.**
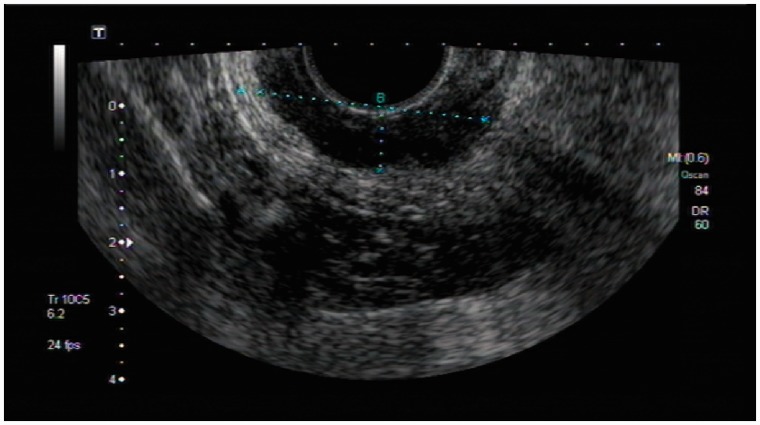
The neoplasm invaded the full-thickness of the intestinal wall, without breaking through the serosa layer; TRUS restaged for uT3N0. Post-operative pathology diagnosed the tumor infiltrated to the muscularis propria and staged for ypT2N0.

The results of comparison between uT-staging and ypT-staging are shown in Table 1. The accuracy of TRUS for T-staging after neo-CRT was 30.9%; the sensitivities for T0, T1, T2, T3, and T4 were 16.3%, 0%, 12.5%, 42.6% and 12.5%, respectively (Fig. 1). There are 60.4% (90/149) cases of over-estimate for T-staging (Fig. 2), and 8.7% (13/149) cases of underestimate.

**Table 1.** Comparison between TRUS for T-staging after neo-CRT and post-operative pathological T-staging

	Pathological T-staging					
uT-staging	ypT0	ypT1	ypT2	ypT3	ypT4	Total
uT0	7	0	1	0	0	8
uT1	0	0	0	3	0	3
uT2	7	1	4	5	0	17
uT3	16	1	14	23	4	58
uT4	13	2	13	23	12	63
Total	43	4	32	54	16	149

The results of comparison between uN-staging and ypN-staging were as follows: TRUS diagnosed 132 cases of uN0 after neo-CRT and 17 cases of uN+, while the post-operative pathology diagnosed 120 cases of ypN0. The diagnostic sensitivity of TRUS for uN0 was 93.3% (112/120), with eight cases misdiagnosed as false positive. The post-operative pathology diagnosed 29 cases of ypN+; thus the diagnostic sensitivity of TRUS for uN+ was 31.0% (9/29), with 20 cases of misdiagnosis. The total accuracy, sensitivity, specificity, positive predictive value and negative predictive value of TRUS for diagnosing the lymph node metastasis after neo-CRT were 81.2%, 31.0%, 93.3%, 52.9% and 84.8%, respectively.

**Fig. 3.**
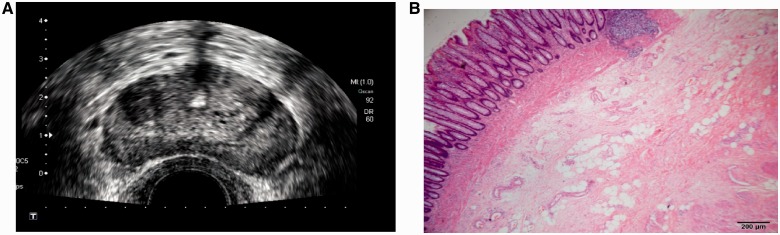
The structure of intestinal wall was complete after neo-CRT, without obvious tumor residual; TRUS staged for pCR (A). Post-operative pathology demonstrated that the tumor was completely regressed, leaving just fibrous tissue without any tumor cells (× 40, hematoxylin-eosin staining) (B).

The results of comparison between TRUS staging and post-operative pathological staging are displayed in Table 2. After combined the T- and N-staging, TRUS staging after neo-CRT were as follows: 5.4% (8/149) cases of uT0N0 (stage u0), 13.4% (20/149) cases of uT1-2N0 (stage uI), 69.8% (104/149) cases of uT3-4N0 (stage uII) and 11.4% (17/149) cases of uN+ (stage uIII). The post-operative pathological staging diagnosed 27.5% (41/149) cases of ypT0N0 (pCR), 21.5% (32/149) cases of ypT1-2N0, 31.5% (47/149) cases of ypT3-4N0 and 19.5% (29/149) cases of ypN+. The sensitivity of TRUS staging for predicting pCR was only 17.1%, but its specificity reached up to 99.1%, with a positive predictive value of 87.5% and a negative predictive value of 75.9% (Figure 3).

**Table 2.** Comparison between TRUS staging after neo-CRT and post-operative pathological staging

	Post-operative pathological staging		
TRUS staging	yp0 (pCR)	ypI-III	Total
u0	7	1	8
uI-III	34	107	141
Total	41	108	149

### The influencing factors for accuracy of TRUS restaging after neo-CRT

The single-factor analysis showed that the restaging accuracy of TRUS for patients with ypT4 and ypN0 were greater (both *P* < 0.001); other clinical factors were not found to be associated with the restaging accuracy of TRUS after neo-CRT (Table 3).

**Table 3.** Analysis of the clinical factors that influence the accuracy of TRUS restaging

Clinical factors	uT-staging			uN-staging		
	Accurate (%)	Inaccurate (%)	*P* values	Accurate (%)	Inaccurate (%)	*P* values
	*n = *46	*n = *103	*n = *120	*n = *29
Pathological T-staging		<0.001				
ypT0	7 (16.3)	36 (83.7)		-	-	
ypT1	0	4 (100)		-	-	
ypT2	4 (12.5)	28 (87.5)		-	-	
ypT3	23 (42.6)	31 (57.4)		-	-	
ypT4	12 (75.0)	4 (25.0)		-	-	
Pathological N-staging						<0.001
N0	-	-		112 (93.3)	8 (6.7)	
N+	-	-		9 (31.0)	20 (69.0)	
Agenda			0.645			0.669
Male	33 (32.0)	70 (68.0)		82 (79.6)	21 (20.4)	
Female	13 (28.3)	33 (71.7)		38 (82.6)	8 (17.4)	
Age (years)			0.071			0.940
≤56	19 (24.4)	59 (75.6)		63 (80.8)	15 (19.2)	
>56	27 (38.0)	44 (62.0)		57 (80.3)	14 (19.7)	
Distance from tumor to anus (cm)			0.241			0.286
<4	8 (22.9)	27 (77.1)		26 (74.3)	9 (25.7)	
≥4	38 (33.3)	76 (66.7)		94 (82.5)	20 (17.5)	
CEA before treatment (ug/L)			0.202			0.597
<5	23 (26.7)	63 (73.3)		68 (79.1)	18 (20.9)	
≥5	23 (36.5)	40 (63.5)		52 (82.5)	11 (17.5)	
Time-point for TRUS after neo-CRT (weeks)			0.604			1.000
<7	41 (29.9)	96 (70.1)		110 (80.3)	27 (19.7)	
≥7	5 (41.7)	7 (58.3)		10 (83.3)	2 (16.7)	

CEA = carcinoembryonic antigen.

## DISCUSSION

Our results show that there are certain limitations for TRUS restaging for locally advanced rectal cancer after neo-CRT. The accuracy of TRUS restaging for T-staging of locally advanced rectal cancer after neo-CRT is relatively low, mainly overestimated for T-staging, mostly for ypT0-2 stage, although the accuracy of T4 stage is greater. The sensitivity of TRUS for negative lymph node (N0 stage) is relatively high, but its sensitivity for diagnosing pCR is low, with a higher specificity and positive predictive value.

TRUS can clearly display the structure of each layer of the intestinal wall, the relationship of lesions with adjacent organs (uterus, ovarian and prostate, etc.), sacral tissue and pelvic wall; thus it is the first choice of T-staging for rectal cancer before treatments. It has been reported that the accuracy (94%) and specificity (86%) are higher for evaluating the local infiltration depth of rectal cancer by transrectal ultrasound endoscopy, but its sensitivity (67%) and specificity (78%) for assessing the lymph node metastasis are not very ideal, which needs to be combined with CT and/or MRI results [6].

There is controversy over the restaging values of TRUS for rectal cancer after neo-CRT. It has been reported that the accuracy of TRUS for T-staging was quite different (this ranged from 38.3–75%), but the accuracy of N-staging was relatively high (from 61–80%), especially for N0 stage with an accuracy of 87% [7–13]. Huh *et al.* reported on 60 patients with locally advanced rectal cancer who underwent TRUS for pre-operative restaging after neo-CRT: the median time interval between neo-CRT and TRUS was 46 (7–90) days [12]. Their results showed that the accuracy of TRUS for T-staging was only 38.3% (36.7% overestimated and 25.0% underestimated), while the accuracy of TRUS for ypT0 was even lower, where none of 10 cases of T0 stage after post-operative pathologic were correctly staged [12]. However, the accuracy was high (81.1%) for TRUS to stage N0 lymph node and 72.6% to stage N+ lymph node; it was much higher when the time interval between the end of neo-CRT and TRUS was no less than 7 weeks (*P* = 0.032) [12]. Vanagunas *et al.* reported that the accuracy of TRUS for T-staging of 82 patients with locally advanced rectal cancer after neo-CRT was 48%, with 38% overestimated (mostly for stage ypT2 of overestimated) and 14% underestimated, while the accuracy of TRUS for lymph node N-staging was 77% [8]. Mezzi *et al.* reported that the accuracies of TRUS in T- and N-staging for 39 patients with locally advanced rectal cancer after neo-CRT were 46% and 69% respectively, with an accuracy of 44% for stage T0-2, 48% for stage T3-4 and 87% for N0 staging [17].

Our results demonstrate that the accuracy of TRUS in T-staging for patients with locally advanced rectal cancer after neo-CRT is relatively low and mainly overestimated, which is in consistent with the literature reports. The overestimated cases are mostly for stage ypT2; 43.8% (14/32) of ypT2 was overestimated as stage uT3 and 40.6% (13/32) was overestimated as stage uT4. The reasons may be that inflammation, edema and fibrosis occur in the tissue surrounding rectal cancer and necrosis occurs in the intestinal wall tissue after neo-CRT, which may damage the normal structure of the intestinal wall. With TRUS it is difficult to distinguish the tumor infiltration from the structure damage of the intestinal wall caused by the above situations. The structural damage to intestinal wall tissue needs some time to heal after exposure to certain doses of radiation and part of it may not be able to heal. Therefore, further research is still needed into whether the time interval between neo-CRT and TRUS examination is related to the accuracy of TRUS.

Huh *et al.* reported that the accuracy of TRUS for T-staging was affected by the tumor location; the accuracy of distance from tumor to anus <4 cm before treatment was relatively high (*P* = 0.049), but no obvious correlation between distance from tumor to anus and the accuracy of TRUS restaging was found in our study [12]. The reason may be associated with bias that was present in patient selection for the former study; for example, their patients withal had low rectal cancers (the distance from tumor to anus being <7 cm), while the distance from tumor to anus of our patients was 1–16 cm [12]. As the tumor does not block the intestinal lumen with the well-prepared bowel, the ultrasonic probe can enter 15 cm into the intestinal lumen and fully detect the rectal neoplasm, which may be one of the reasons for an absence of an obvious correlation between distance from tumor to anal verge and the accuracy of TRUS restaging in this study.

In addition, our results show that the accuracy of TRUS for N-staging is high. The accuracy of TRUS for N0 staging reached up to 84.8%, which is consistent with the literature reports, but its sensitivity for positive lymph nodes was relatively low (only 31.0%) [7–13]. At present, the evaluation criteria for diagnosing positive lymph nodes with metastasis is based mainly on the sizes of lymph nodes. However, it has been reported that 48% (96/200) of the metastasis lymph nodes cleaned by the radical resection of rectal carcinoma with a diameter of <5 mm, and the tiny lymph node metastasis rate, was higher with the increasing of T-staging, which may lead to the lower accuracy of ultrasound for assessing whether a lymph node occurs metastasis [18].

There have been few studies concerning the accuracy of TRUS for diagnosing pCR. Radovanovic *et al.* studied TRUS restaging in 44 patients and found that the sensitivity of TRUS for pCR was 20%; TRUS only correctly diagnosed one in five cases of post-operative pCR [11]. Our result of TRUS sensitivity in diagnosing pCR was only 17.1%, which is in accordance with that of Radovanovic *et al.* and the reason may be attributed to the lower sensitivity of TRUS for T0 stage (16.3%) [11]. The prognosis of patients with pCR is good, and many researchers believe that patients with pCR after neo-CRT can accept local excision or close follow-up treatment strategy in order to avoid the complications and *sequela* of radical surgery [4, 5, 19]. Therefore, it is very important to screen out pCR pre-operatively in the clinic. Although the sensitivity of TRUS for diagnosing pCR is relatively low in our study, 87.5% (7/8) of pCRs were correctly diagnosed by TRUS, and only one case was post-operatively, pathologically staged for ypT2N0. Our results suggest that the specificity and positive predictive value of TRUS for diagnosing pCR are high, and there is still a certain value in TRUS for screening pCR.

There are some limitations in this research. First, this is a retrospective study in which information on ultrasonic staging can only be obtained according to imaging data and description at the TRUS after neo-CRT. Second, there is a steep learning curve for transrectal ultrasound operation techniques, and different inspectors have different diagnostic criteria, so its accuracy is, to a certain extent, governed by different operators' experience. Third, the sample size of this research is relatively small.

In conclusion, the accuracy of TRUS uT-staging for patients with locally advanced rectal cancer after neo-CRT is relatively low, mainly shown by overestimates for ypT2 stage, but its sensitivity for ypT4 stage is greater. The accuracy of TRUS for negative lymph node N0 staging is high. Although the sensitivity of TRUS for diagnosing pCR is low, the specificity and positive predictive value are high, which demonstrates that it is valuable for screening patients with pCR. The value of TRUS restaging for patients with rectal cancer after neo-CRT still needs further research.

## FUNDING

This study was supported by National Natural Science Funding of China (81071891, 81172209) and Guangdong Provincial Science & Technology Funding (2010B0807017, 2010B031600090).

**Conflict of interest:** none declared.
